# Virucidal efficacy of glutaraldehyde for instrument disinfection

**DOI:** 10.3205/dgkh000369

**Published:** 2020-12-14

**Authors:** Florian H.H. Brill, Britta Becker, Daniel Todt, Eike Steinmann, Joerg Steinmann, Dajana Paulmann, Birte Bischoff, Jochen Steinmann

**Affiliations:** 1 Dr. Brill + Partner GmbH Institute for Hygiene and Microbiology, Bremen, Germany; 2 Department for Molecular & Medical Virology, Ruhr-University Bochum, Bochum, Germany; 3 Institute of Clinical Hygiene, Medical Microbiology and Infectiology, Paracelsus Medical University, General Hospital Nürnberg, Nuremberg, Germany; 4 Institute of Medical Microbiology, University Hospital Essen, University of Duisburg-Essen, Essen, Germany

**Keywords:** glutaraldehyde, virucidal efficacy, instrument disinfection

## Abstract

**Aim:** Glutaraldehyde (GDA) is an active ingredient in many instrument disinfectants and is effective against a broad spectrum of microorganisms. In the past, the virus-inactivating properties of these products were mainly claimed based on quantitative suspension tests with different test viruses. Recently, however, a European Norm EN 17111:2018 has been published which allows examination of instrument disinfectants in a surface carrier test, simulating practical conditions. Therefore, it is of interest to evaluate GDA for the ability to inactivate the viruses used in this European Norm as test viruses.

**Methods:** The virucidal efficacy of GDA as the active ingredient in instrument disinfectants was evaluated with 4 different test viruses in a method simulating practical conditions (EN 17111:2018).

**Results:** With a fixed exposure time of five minutes at 20°C, 100 ppm GDA were necessary to inactivate vaccinia virus, classifying it as a limited spectrum virucidal activity for pre-cleaning products. For adenovirus, 125 ppm GDA were required, whereas for murine norovirus as a surrogate for human norovirus, 4,000 ppm GDA were required for a significant reduction of viral titres. Both non-enveloped viruses must be tested to prove virucidal activity in EN 17111:2018. But even 4,000 ppm were not enough to yield a 4 log_10_ reduction of the murine parvovirus at 20°C. This virus is only required as a test virus using this method if temperatures ≥40°C are used.

**Conclusion:** GDA, as the active ingredient of many instrument disinfectants, shows virucidal efficacy at 20°C. The necessary concentrations are strongly dependent on the stability of the test viruses. Due to the high stability of murine norovirus, GDA levels of 4,000 ppm were required to inactivate this virus within the 5-minute exposure time.

## Zusammenfassung

**Zielsetzung:** Glutaraldehyd (GDA) besitzt ein breites Wirkspektrum gegen Mikroorganismen und wird daher in vielen Mitteln zur Instrumentendesinfektion verwendet. In der Vergangenheit wurden virusinaktivierende Eigenschaften dieser Produkte hauptsächlich auf Grundlage quantitativer Suspensionstests mit verschiedenen Testviren ermittelt. Inzwischen wurde die europäische Norm EN 17111:2018 veröffentlicht, welche die Untersuchung der Instrumentendesinfektionsmittel in einem Oberflächenträgertest unter praktischen Bedingungen ermöglicht. Daher ist es von Interesse, diesen wichtigen Wirkstoff vieler Instrumentendesinfektionsmittel auf die Fähigkeit zur Inaktivierung von Viren zu untersuchen, die in dieser europäischen Norm als Testviren ausgewählt wurden.

**Methode:** Die viruzide Wirksamkeit von GDA als Wirkstoff in Instrumentendesinfektionsmitteln wurde mit 4 verschiedenen Testviren in einer Methode mit Simulation praktischer Bedingungen überprüft (EN 17111:2018).

**Ergebnisse:** Die Ergebnisse der Studie mit einer festen Expositionszeit von fünf Minuten bei 20°C zeigten, dass 100 ppm GDA für die Inaktivierung des Vaccinia-Virus erforderlich waren. Daraus resultiert die Einstufung als „begrenzt viruzid“ für Vorreinigungsprodukte. Für das Adenovirus wurden 125 ppm GDA benötigt, während für das murine Norovirus als Surrogat für das humane Norovirus 4.000 ppm GDA für eine signifikante Verringerung der Virustiter erforderlich waren. Beide nicht umhüllten Viren müssen gemäß EN 17111:2018 getestet werden, um den Nachweis einer viruziden Aktivität zu erbringen. Aber selbst 4.000 ppm reichten nicht aus, um eine Reduktion des murinen Parvovirus um 4 log_10_ bei 20°C nachzuweisen. Dieses Virus wird mit dieser Methode aber nur bei Temperaturen ≥40°C eingesetzt.

**Fazit:** Zusammenfassend lässt sich sagen, dass GDA als Wirkstoff vieler Instrumentendesinfektionsmittel eine viruzide Wirksamkeit bei 20°C besitzt. Die notwendigen Konzentrationen hängen stark von der Stabilität der Testviren ab. GDA- Konzentrationen von 4.000 ppm bei einer 5-Minuten Einwirkzeit sind erforderlich, bedingt durch die ausgeprägte Stabilität des murinen Norovirus.

## Introduction

Glutaraldehyde (GDA) has long been the active ingredient in many high-level instrument disinfectants for semi-critical devices or instruments. An aqueous 2% alkaline GDA solution is one of the most common disinfectants for endoscope decontamination in hospitals.

In the past, the virucidal activity of GDA was mainly examined against stable viruses from the *Picornaviridae* family in a quantitative suspension test describing the virucidal activity under various test conditions [1], [2], [3]. The European Norm (EN) 14476 [4] or the Guideline of *Deutsche Gesellschaft zur Bekämpfung der Viruskrankheiten e.V.* (DVV) and the Robert Koch Institute (RKI) [5] allow the comparison of different instrument disinfectants with many test viruses in suspension tests under well-defined conditions. In these tests, a disinfectant (8 parts) is mixed with a protein load (1 part) and a test virus suspension (1 part), including various controls. At the end of a fixed exposure time. Residual virus titres are determined and the reduction factor (RF) is calculated. But this *in vitro* approach does not describe the situation under practical, clinical conditions.

With the recent introduction of EN 17111:2018, a test simulating practical conditions is now available, thus allowing evaluation of the virucidal efficacy of instrument disinfectants [6]. In this test, a test virus suspension mixed with bovine serum albumin (BSA) is spread on a glass carrier. After drying, the glass carrier is immersed in a chosen concentration of the instrument disinfectant for a fixed exposure time in parallel with a control with water of standardized hardness. After the chosen exposure time, the carriers are transferred into a container with Minimum Essential Medium together with glass beads. Immediately after elution, the non-inactivated viruses are determined by endpoint dilution. In this study, we evaluated the virucidal activity of GDA with the 4 different test viruses of EN 17111:2018 using this method simulating practical conditions.

## Materials and methods

A carrier assay using frosted glass based on EN 17111:2018 was run with a fixed exposure time of five minutes and a test temperature of 20°C with modified vaccinia virus Ankara (ATCC VR-1508) (MVA) for pre-cleaning products only (classification: limited-spectrum virucidal activity). The MVA was obtained from Dr. Manteufel, Leipzig, Germany.

*BHK 21-cells* (passage 108) originated from the Friedrich Löffler Institute (FLI), Greifswald, Germany. For preparation of test virus suspension, *BHK 21-cells* were cultivated using Eagle’s Minimum Essential Medium with Hank’s BSS and 10% or 2% fetal calf serum (FCS).

Adenovirus type 5 strain Adenoid 75 (ATCC VR-5) (AdV) and murine norovirus strain S99 Berlin (MNV) were chosen for instrument disinfection. Before the inactivation assays, the adenovirus was passaged 3 times in *A549 cells*. The *A549 cells* (passage 128) were obtained from Vircell, S.L., Spain.

The murine norovirus (MNV) (passage 3) was obtained from the FLI, Germany. MNV was passaged in *RAW 264.7 cells* (murine macrophage cell line, ATCC TIB-71). *RAW 264.7 cells* were cultured with Dulbecco’s Modified Eagle’s Medium with 4.5 g/l glucose and 10% FCS with low endotoxin.

The murine parvovirus, minute virus of mice (MVM), strain Crawford (ATCC VR 1346) was included in this study, which should only be used for temperatures ≥40°C in EN 17111:2018. MVM (passage 4) was obtained from the Paul Ehrlich Institute, Langen, Germany. *A9 cells* (mouse cell line, Paul Ehrlich Institute) (passage 30) were cultivated in a 175 cm^2^ flask with DMEM and 10% FCS. The GDA solution (CAS Number 111-30-8) was obtained from Sigma-Aldrich-Chemie GmbH [25%].

All details of this quantitative carrier test are described in the EN 17111:2018. The surface-sandblasted frosted glass carriers were prepared as follows. One volume of 0.3% BSA solution was mixed with nine volumes of test virus suspension. In brief, 50 µL of this virus inoculum were pipetted onto the inoculation square of the carrier, followed by drying. The glass carrier with the dried virus inoculum was placed in a 30-mL cylindrical screw tube containing 10 mL of the GDA solution. Due to existing data with GDA-based instrument disinfectants, different concentrations were tested against the 4 test viruses. At the end of the exposure time, the carriers were transferred into a second screw tube with MEM and glass beads, followed by mixing for 60 s. Infectivity was stopped by immediate serial dilution (1:10 dilutions) with ice-cold MEM. Next, 100 µL of each dilution were placed in eight wells of a sterile polystyrene flat-bottomed 96-well microtitre plate containing 100 µL cell suspension. Titre reduction is shown as the difference between the virus titre after exposure time with different GDA concentrations and the virus titre with hard water. In parallel, tests were run without any virus as control for cytotoxicity.

The virus titres were determined using the method of Spearman [7] and Kaerber [8] and expressed as log_10_TCID_50_/mL with a 95% confidence interval. A reduction of infectivity of ≥4 log_10_ steps (inactivation ≥99.99%, RF≥4) is regarded in the EN 17111:2018 as evidence of virucidal efficacy [6].

## Results

**Figure 1:**
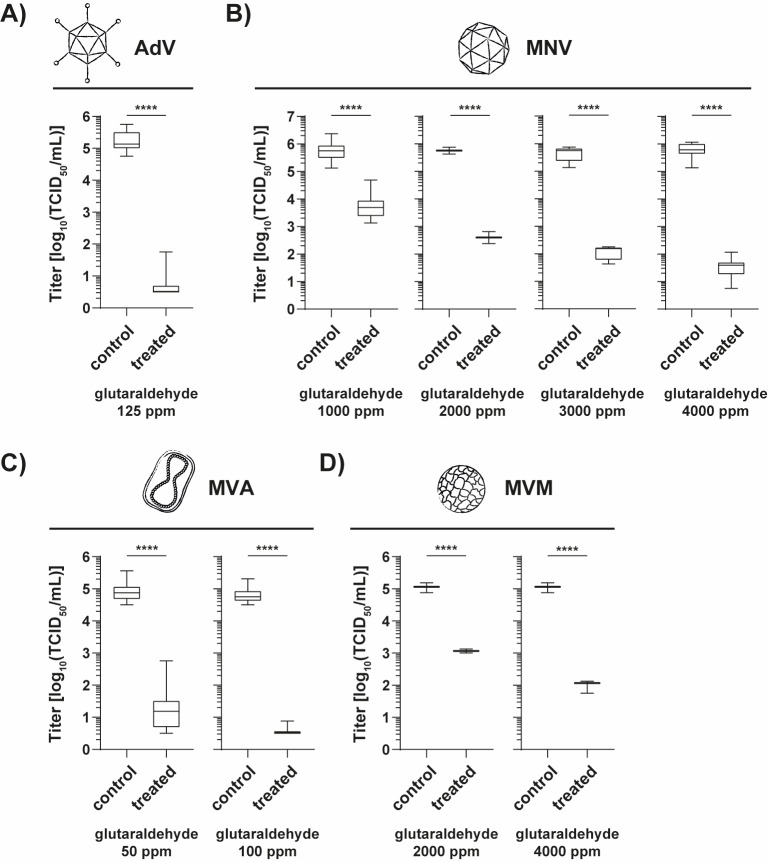
Inactivation of adenovirus type 5 (AdV), murine norovirus (MNV), modified vaccinia virus Ankara (MVA), and minute virus of mice (MVM), a murine parvovirus, by different glutaraldehyde concentrations at 20°C under clean conditions according to EN 17111:2018. The decrease in virus titre is shown in comparison to the hard water control. Five minutes were chosen as the fixed exposure time.

The virucidal efficacy of GDA as the active ingredient in instrument disinfectants was evaluated with 4 different test viruses in a method simulating practical conditions. The results are given in Figure 1 (Fig. 1). The GDA concentrations were chosen with respect to the different stability of the test viruses. The enveloped MVA was the most fragile virus. Even 100 ppm were able to achieve a RF of ≥4.24±0.22 (Figure 1C (Fig. 1)), qualifying it as efficacious for pre-cleaning products according to EN 17111:2018. For AdV, the tested 125 ppm GDA resulted in a RF of ≥4.54±0.43 (Figure 1A (Fig. 1)). A higher stability was observed for MNV. Here, 1000, 2000 and 3000 ppm GDA were not enough to reduce virus titre over four orders of magnitude. Finally, 4000 ppm GDA produced a titre decrease of ≥4.26±0.40 log10 steps (Figure 1B (Fig. 1)) after the 5-minute exposure time. In the case of MVM testing with 4,000 ppm GDA, only a reduction below the requested 4-log threshold (RF=3.06±0.29) was achieved (Figure 1D (Fig. 1)).

## Discussion

By performing EN 17111:2018, the virus-inactivating properties of instrument disinfectants can be tested with a method simulating practical conditions [6]. MVA, AdV, MNV and MVM (for elevated temperature only) are the test viruses used here. MVA is chosen as test virus reference for all enveloped viruses, resulting in classification as “limited-spectrum virucidal activity” for all enveloped viruses for pre-cleaning products. This classification is important when important enveloped blood-borne viruses, e.g, hepatitis B virus and hepatitis C virus, are to be inactivated during the cleaning step. According to EN 17111:2018, AdV and MNV are test viruses for virucidal efficacy below 40°C, whereas MVM is only used with elevated temperatures (≥40°C) due to the great thermostability of this virus. Furthermore, it is important to know that a virucidal claim of instrument disinfectants can only be given based on EN 17111:2018 after passing the requirements of EN 14476:2019 for AdV, MNV and poliovirus [6]. Poliovirus is not the test virus in tests simulating practical conditions, because infectivity is significantly decreased after the drying process [9].

For GDA as the fixed reference in EN 17111:2018, the chemical and physical parameters are described in detail in the European Norm. However, no data for the concentration used as the reference are available to date. Therefore, our data should additionally help determine exact reference concentrations. As expected, MVA – representing all enveloped viruses – was the most fragile virus. 100 ppm was enough for viral inactivation, whereas 50 ppm GDA failed. Interestingly, a great difference regarding the stability of both non-enveloped viruses was observed. MNV (4,000 ppm) required much higher concentrations than did AdV (125 ppm), demonstrating a distinctively greater stability towards GDA in the test simulating practical conditions.

In a previous test with practical conditions testing peracetic acid (PAA), MNV was also more stable than AdV. 400 ppm PAA at 20°C was enough for a reduction of 4 log_10_ steps with AdV, whereas MNV inactivation required 1,000 ppm PAA at 20°C with five minutes’ exposure time [10].

Some surface disinfectants used in the healthcare setting are also based on GDA. Therefore, it might be interesting to compare results of instrument tests where carriers are immersed as shown here to results of carrier tests overlying the virus suspension. With a test based on stainless steel discs for surface disinfectants, it was shown that concentrations between 125 and 500 ppm GDA were necessary for AdV inactivation within a 5-minute exposure time [11], whereas 125 ppm in the instrument test were required. For MVM, 2,500 ppm GDA were enough to produce a 4-log_10_ reduction of virus titres in the surface test, whereas in our study on instrument disinfectants, 4,000 ppm GDA failed to produce such an inactivation (RF=3.06±0.29). This means that results from the instrument test EN 17111:2018 cannot be transferred to surface disinfectants, which must be tested in a different way.

In summary, our data will help to evaluate GDA-based instrument disinfectants often used for manual cleaning for their efficacy to inactivate enveloped and non-enveloped viruses, which are now described as test viruses in the recently published EN 17111:2018 [6]. Furthermore, our results will help establish reference values for this European Norm.

## Notes

### Conflict of interest

JS works as a consultant for Dr. Brill + Partner GmbH Institute for Hygiene and Microbiology. The other authors declare that they have no competing interests.

### Authors’ contributions

FB, JoeS, ES and JS formulated the study questions and designed the study. BB, DP and BB were responsible performing all experimental data. DT was responsible for data evaluation. All authors read and approved the final manuscript.
